# Clinical Presentation of a Patient With Tuberculous Myocarditis: Case Report and Review of Literature

**DOI:** 10.7759/cureus.22715

**Published:** 2022-02-28

**Authors:** Vamsidhar Vennamaneni, Farah Chohan, Pedram Rad, James Rodriguez, Ravi Gupta, George Michel

**Affiliations:** 1 Internal Medicine, Larkin Community Hospital, South Miami, USA; 2 Research and Academic Affairs, Larkin Community Hospital, South Miami, USA; 3 Medical School, Ross University School of Medicine, Miramar, USA

**Keywords:** complications of tb, morbidity and mortality, acute decompensated heart failure, tuberculous myocarditis, miliary tuberculosis

## Abstract

Tuberculous myocarditis has a high mortality rate and is often associated with a delay in the diagnosis because of the low index of suspicion and insidious course. Most of the reported cases predominantly occur in young, immunocompetent patients. Delays in diagnosis may result in fatal complications. Through this case report, we aim to shed light on some of the clinical features of tuberculous myocarditis and promote a high index of suspicion for early diagnosis and timely management.

## Introduction

Although tuberculosis is primarily a pulmonary disease, however, it may involve other extrapulmonary organs as well including the heart. Isolated myocardial involvement in tuberculosis is exceedingly rare; several reports suggest that it can present with atrioventricular block, ventricular arrhythmias, congestive cardiac failure, or sudden cardiac death [1]. Tuberculous myocarditis is associated with high mortality and is often diagnosed during post-mortem examination because of the low index of suspicion and insidious course. Most of the reported cases are predominantly in young, immunocompetent patients under 45 years of age [2]. We aim to discuss some of the clinical features of this condition and promote a high index of suspicion for early diagnosis, and thereby timely management of tuberculous myocarditis.

## Case presentation

**Figure 1:**
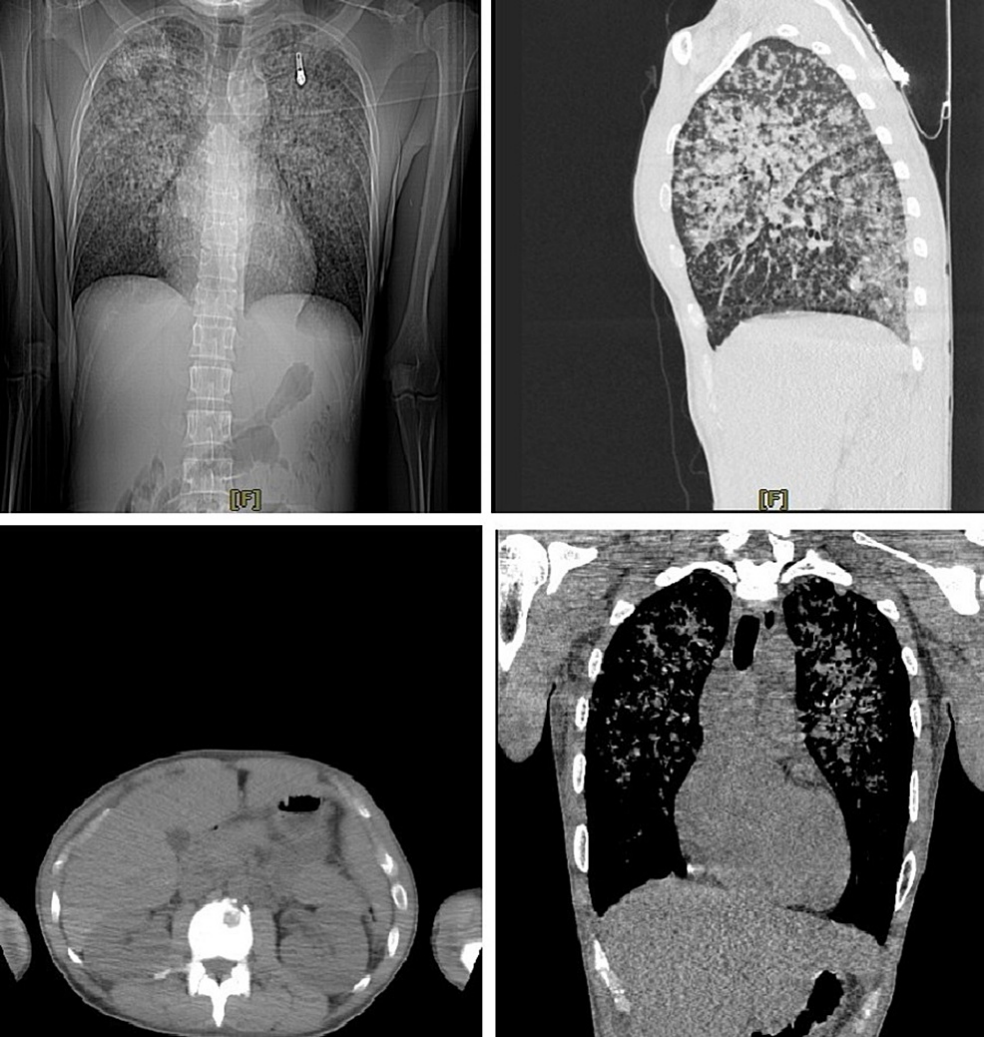
CT scan of the chest as seen from different angles. Numerous bronchovascular distribution of nodules, worse in upper central lungs, can be observed. An osteolytic lesion can also be seen in the lower left quadrant.

The patient was a 29-year-old Asian male cruise line worker who had shortness of breath and dry cough for one week before presenting to the Emergency Department (ED). The patient reported episodes of fever along with pleuritic type chest pain. He also complained of weight loss, but was unsure of how much as he never weighed himself. He denied nausea, vomiting, night sweats, dysuria, diarrhea, or hemoptysis. He had no previous medical history or known exposure to tuberculosis. In the ED, laboratory tests showed hemoglobin: 9.5, hematocrit: 28.4, sodium: 128, aspartate aminotransferase: 108, alanine aminotransferase: 54, and alkaline phosphatase: 201. The patient was admitted to the medical floor and put on droplet precautions. On day one of admission, the saturation was 90-91% and 2L oxygen via nasal cannula was given as needed. Chest CT from admission revealed numerous bronchovascular distribution of nodules that were worse in the upper central lungs. An osteolytic lesion was also spotted on the vertebral body (Figure 1).

**Figure 2:**
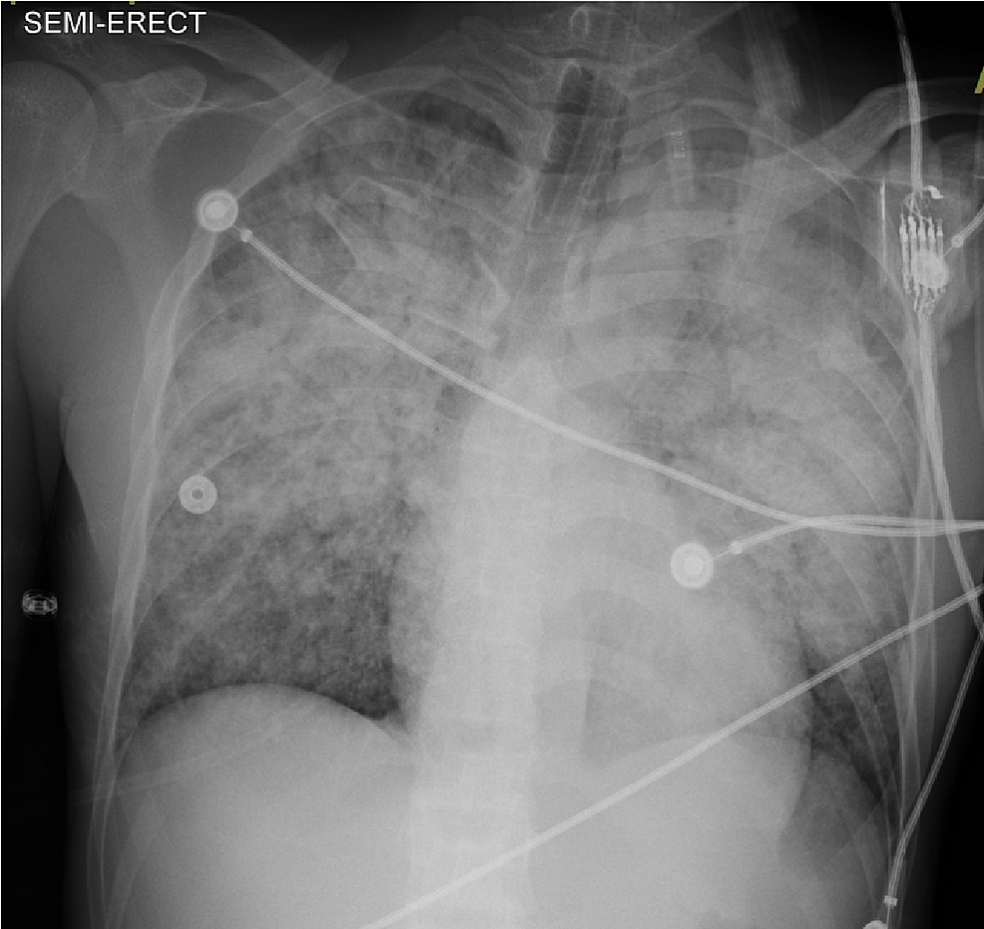
Chest x-ray showing diffuse bilateral opacities in the upper lungs.

In suspicion of tuberculosis, the sputum sample was sent for acid-fast bacillus (AFB) tests and blood cultures were also sent. The ultrasound abdomen showed a rounded hypoechogenicity visualized at the pancreatic neck with no significant color doppler flow. Empiric antibiotic therapy was started with ceftriaxone and azithromycin. On the third day of admission, the patient underwent bronchoscopy and a bronchoalveolar lavage was obtained for AFB smear/culture, *Mycobacterium tuberculosis* PCR, nucleic acid amplification test (NAAT) per Infectious Diseases Society of America/American Thoracic Society (IDSA/ATS) guidelines. Overnight, the patient developed acute hypoxia, tachypnea, and tachycardia. The respiratory rate was in the range of 40-50, heart rate was 130-145 beats per minute, and he was saturating at 75% on non-rebreather mask. The patient was unable to talk in full sentences and demonstrated use of accessory muscles of respiration as well as gasping gestures. The decision was made to emergently intubate the patient and place him on mechanical ventilation. Upon successful intubation, the patient was found to be severely hypotensive with mean arterial pressure (MAP) below 65. Despite administration of 5L of crystalloids for volume resuscitation, the patient remained hypotensive and the decision was made to place an internal jugular central venous catheter for administration of vasoactive medications. The patient tolerated the procedure without any difficulties. He continued to be hypoxic while ventilated requiring high positive end-expiratory pressure (PEEP) and an inverse ratio of 3:1 to maintain oxygenation. Norepinephrine was initiated to maintain his MAP >65. Arterial blood gases (ABGs) obtained at the time of intubation demonstrated a respiratory alkalosis with compensated metabolic acidosis as well as significant hypoxemia despite the near 100% fraction of inspired oxygen (FiO2), pH: 6.96, pCO2: 76.8, pO2: 78.2, and HCO3: 17.4. Preliminary sputum culture was positive for gram (+) cocci in pairs. HIV serology was negative. Additional cultures and serology were collected, including cryptococcal antigen, histoplasma urine antigen, blastomycosis urine antigen, coccidioides antibody, aspergillosis antigen, cytomegalovirus culture, fungal culture. Liver enzymes continued to trend up through the day. According to the infectious disease team's recommendations, ceftriaxone and azithromycin were discontinued and the patient was started on intravenous vancomycin, meropenem, and voriconazole. The AFB smear/culture came back positive for *Mycobacterium tuberculosis*. A chest x-ray done at that time showed diffuse bilateral opacities that were slightly worsened in the upper lungs (Figure 2).

Bedside echocardiography showed a left ventricular ejection fraction (LVEF) of 10% with left ventricular borderline dilatation and significant regional wall motion abnormalities. There was global hypokinesis of the left ventricle with global thinning of the left ventricular walls. Doming of the anterior mitral valve leaflet was also present. Despite the resuscitative measures, the patient’s condition continued to deteriorate and the patient died after a code blue according to the Advanced Cardiovascular Life Support (ACLS) protocol.

## Discussion

Three different types of tuberculous myocarditis have been previously reported, which include diffuse infiltrative, miliary, and nodular with central caseation. Tuberculous myocarditis can sometimes remain clinically asymptomatic or it can present with ventricular arrhythmias, sudden cardiac death, long QT syndrome, heart block, or congestive heart failure. Sudden cardiac death related to tuberculosis is also being increasingly reported. Cardiac complications occur rarely but they are potentially treatable, and early recognition of the underlying cause is key to timely management [1]. Michira et al. conducted a systematic review of cases including tuberculous myocarditis and pericarditis and found that in 81% of the cases, tuberculous myocarditis was reported in immunocompetent patients below 45 years of age. The ratio of males to females affected with tuberculous myocarditis was 2:1. The left ventricle was most commonly affected. Their study revealed that although most cases responded to anti-tuberculosis drug therapy, the risk of sudden cardiac death from this condition still persisted despite the treatment, hence pointing towards the significance of close monitoring and symptomatic relief of cardiac abnormalities [2].

In another case report published by Cowley et al., the patient was diagnosed at post-mortem examination with myocardial tuberculosis leading to acute decompensated heart failure. The patient developed severe hypoxia with evidence of severe biventricular failure on echocardiography and continued to deteriorate [3]. According to a study published by Lopez et al., the authors studied articles published between 1955 to 2020 and found that the frequency for cardiac involvement in tuberculosis cases was pericarditis in 2-5% of the cases, myocarditis in 0.14-2% of the cases, and aortitis in 0.3% of the total cases diagnosed with tuberculosis. Clinical manifestations of tuberculous myocarditis include disturbances of the conduction system, ventricular fibrillation, or cardiac arrest. Symptoms of heart failure appear when the vegetations result in a hemodynamic compromise leading to severe valve insufficiencies [4].

In a case report published by Choudhary et al., a 34-year-old patient with disseminated tuberculosis developed severe left ventricular systolic dysfunction accompanied by an increase in cardiac biomarkers, severe left ventricular regional wall hypokinesis with a markedly reduced ejection fraction of up to 25-30%, bilateral upper and middle lobe ground-glass opacities, and mediastinal and hilar lymphadenopathies. She was started on anti-tuberculosis therapy, beta-blocker, angiotensin-converting enzyme inhibitor, and corticosteroids. She was subsequently discharged after one week with significant improvement in her clinical status [5].

## Conclusions

From the existing literature, it is quite evident that tuberculous myocarditis can lead to a rapid decline of cardiac function, and may prove to be fatal. Clinicians should have a a high index of suspicion in order to diagnose this condition in a timely manner and for a prompt management. Delays in diagnosis may result in fatal complications.
